# The Role of Climatic and Density Dependent Factors in Shaping Mosquito Population Dynamics: The Case of *Culex pipiens* in Northwestern Italy

**DOI:** 10.1371/journal.pone.0154018

**Published:** 2016-04-22

**Authors:** Giovanni Marini, Piero Poletti, Mario Giacobini, Andrea Pugliese, Stefano Merler, Roberto Rosà

**Affiliations:** 1 Department of Mathematics, University of Trento, Trento, Italy; 2 Department of Biodiversity and Molecular Ecology, Research and Innovation Centre, Fondazione Edmund Mach, San Michele all'Adige (TN), Italy; 3 Bruno Kessler Foundation, Trento, Italy; 4 Dondena Centre for Research on Social Dynamics and Public Policy, Department of Policy Analysis and Public Management, Universitá Commerciale L. Bocconi, Milan, Italy; 5 Department of Veterinary Sciences, University of Torino, Torino, Italy; National Taiwan Ocean University, TAIWAN

## Abstract

*Culex pipiens* mosquito is a species widely spread across Europe and represents a competent vector for many arboviruses such as West Nile virus (WNV), which has been recently circulating in many European countries, causing hundreds of human cases. In order to identify the main determinants of the high heterogeneity in *Cx*. *pipiens* abundance observed in Piedmont region (Northwestern Italy) among different seasons, we developed a density-dependent stochastic model that takes explicitly into account the role played by temperature, which affects both developmental and mortality rates of different life stages. The model was calibrated with a Markov chain Monte Carlo approach exploring the likelihood of recorded capture data gathered in the study area from 2000 to 2011; in this way, we disentangled the role played by different seasonal eco-climatic factors in shaping the vector abundance. Illustrative simulations have been performed to forecast likely changes if temperature or density–dependent inputs would change. Our analysis suggests that inter-seasonal differences in the mosquito dynamics are largely driven by different temporal patterns of temperature and seasonal-specific larval carrying capacities. Specifically, high temperatures during early spring hasten the onset of the breeding season and increase population abundance in that period, while, high temperatures during the summer can decrease population size by increasing adult mortality. Higher densities of adult mosquitoes are associated with higher larval carrying capacities, which are positively correlated with spring precipitations. Finally, an increase in larval carrying capacity is expected to proportionally increase adult mosquito abundance.

## Introduction

Zoonotic pathogens are believed to cause about three quarters of human emerging infectious diseases, many of which (22%) are spread by vectors such as mosquitoes [1]. One of the most recent emerging mosquito-borne diseases in the Western Hemisphere is West Nile Virus (WNV), a flavivirus first isolated in Uganda in 1937 [2]. It is maintained in a bird-mosquito transmission cycle primarily involving *Culex* species mosquitoes of which the *Cx*. *pipiens* complex is thought to be one of the most important in Europe [3]. In recent years, WNV has been circulating in many European countries, including Italy, causing hundreds of human cases [4]. *Cx*. *pipiens* is also involved in the transmission of other human and animal pathogens such as Usutu virus [5], whose first case outside Africa was recorded in Italy in 2009 [6], St. Louis encephalitis [7], which caused about a hundred human cases in the US during the last decade [8], Rift Valley fever [9], Sindbis virus [10], avian malaria and filarial worms [11].

The transmission of mosquito-borne diseases is largely driven by the abundance of the vector [12, 13]. Thus, rigorous surveillance of mosquito density and control programs based on its reduction represent key components of disease containment and prevention. Therefore, in order to design appropriate control strategies it is crucial to understand the population dynamics of existing vector populations and evaluate how it depends on environmental factors.

In the Piedmont region of Northwestern Italy, an extensive program of monitoring adult mosquitoes has been implemented, since 1997, by the Municipality of Casale Monferrato and the Istituto per le Piante da Legno e l’Ambiente (IPLA). The area is at risk for WNV, because of the presence of suitable vector and reservoir host populations, and the increasing numbers of human cases of WNV in adjacent areas [14, 15]. Previous studies [16, 17] analyzed spatio-temporal variations of mosquito species collected in the area, detecting a very high heterogeneity in the temporal pattern of mosquito population dynamics both inter- and intra-annually. In particular, looking at *Cx*. *pipiens* population dynamics from 2001 to 2011, Rosà and co-authors [17], detected a huge variation in total yearly mosquito abundance among different traps, ranging from 40 to more than 4000 individuals captured per year. Also the timing of mosquito seasonal dynamics varied significantly among traps and years. Specifically, for around 90% of the observations the start of mosquito season varied from the beginning of June to mid-July, while the length of mosquito season varied from 45 to 90 days [17].

The main goal of our work is to describe and interpret in a robust theoretical framework the high heterogeneity observed among different seasons for *Cx*. *pipiens* population dynamics in Northwestern Italy [17], by explicitly taking into account some important eco-climatic and biological factors.

In their work, Rosà and co-authors [17] found that precipitation and temperatures during the early period of the year (spring and early summer) might remarkably influence *Cx*. *pipiens* population dynamics. In particular, warm temperatures early in the year were associated with an earlier start of the mosquito season and increased season length, while early precipitation delayed the start, and shortened the length of the mosquito season, but increased total abundance [17]. Indeed, temperature is well known to affect several aspects of *Cx*. *pipiens* life cycle including development and survival rates [18, 19].

Density-dependence in mosquito population growth is another important factor in regulating *Cx*. *pipiens* population dynamics [20]. In fact, it has been found that inclusion of density-dependence, in combination with key environmental factors, significantly improves model prediction of *Cx*. *pipiens* population expansion in Northern Italy [20]. By using a statistical model, the authors found that the most significant environmental drivers of *Cx*. *pipiens* population dynamics were the daylight duration and temperature conditions in the 15 day period prior to sampling while precipitation and humidity had only a minor influence on *Cx*. *pipiens* growth rates.

Diapause is a common mechanism adopted by mosquitoes to survive through winter. While other mosquitoes, for instance *Aedes albopictus*, overwinter through diapausing eggs [21], in the case of *Cx*. *pipiens*, only adult females undergo diapause halting blood feeding and therefore host-seeking behavior [21]. More specifically, immature stages develop into diapausing adults according to the photoperiod they are exposed to [22].

We therefore develop a density-dependent stochastic model that describes temporal variations of *Cx*. *pipiens* population dynamics including the effect of temperature and daylight duration on the abundance of both adults and immature stages of *Cx*. *pipiens*. Mechanistic models include, with more or less details, the biological processes driving mosquito population dynamics and provide a suitable framework to investigate the main determinants of dynamical patterns beyond the observed conditions [23]. Several mechanistic models have been proposed to explore mosquito population dynamics especially for *Anopheles* species (e.g. [24–27]) and *Aedes albopictus* (e.g. [28–30]) while, to the best of our knowledge, fewer attempts have been carried out for modelling *Cx*. species population dynamics [31–34]. Mathematical models represent a powerful tool to investigate the role played by different climatic factors on vector population dynamics and to evaluate the effectiveness of alternative mosquito control strategies, as suggested by recent works on *Cx*. *quinquefasciatus* [33], *Anopheles species* [27] and *Aedes albopictus* [30].

We follow a stochastic approach as deterministic models ignore the contribution of demographic stochasticity which is especially relevant when the vector population is low, for instance at the beginning and at the end of mosquito activity season. The proposed model explicitly accounts for the temporal variation of all immature stages, i.e. eggs, four larval instars and the pupal stage; it is assumed that the lengths of all mosquito life stages depend on temperature and that developmental rates of larval stages are density-dependent; finally, a diapausing mechanism is included in response to the photoperiod.

The effect of precipitation on survival and development of mosquito life stages is not explicitly accounted for, as, to the best of our knowledge, no reliable data on *Cx*. *pipiens* are present in literature for modeling and calibrating such mechanism. In the Results Section, we discuss correlation of density dependence with precipitation, which could indirectly enter the model in this way.

Finally, extensive model simulations have been carried out in order to better understand the role played by different eco-climatic factors in shaping the seasonal specific vector dynamics and to forecast, under various illustrative scenarios, likely changes in *Cx*. *pipiens* seasonal dynamics if temperature or density–dependent inputs would change.

## Methods

### Data

*Cx*. *pipiens* mosquitoes were collected on public land using CO_2_ dry ice baited traps operated by Municipality of Casale Monferrato and the Istituto per le Piante da Legno e l’Ambiente (IPLA), under the regional program for mosquito surveillance, authorized by Regione Piemonte. The traps were dispersed over an area of 987 km^2^ in the Eastern Piedmont Region in North West of Italy (see Figure G in S1 Text and [16, 17] for more details). The study region is characterized by cold winters and warm summers (average temperature of 0.4°C and 24°C, respectively), abundant precipitation (~600 mm/yr) and by a mostly agricultural landscape (86%) with few urban settlements (3%). This makes the area a highly suitable habitat for *Cx*. *pipiens*. Traps were set up one night every week, for a twenty-week period starting at the beginning of May and ending in mid-September, for 12 consecutive years (2000–2011). Traps were collected the following day and all catches counted, sexed and identified. Since some locations were not deployed every year [16, 17], we consider in this study only data coming from traps sampled for all the 12 consecutive years (i.e., 24 out of 44). Trapping conditions including positioning, battery and trap type, and CO2 source (0.5 kg placed in traps each evening before a capture session) were identical among different sites and years [17]. Moreover, during the study period, no relevant activities were performed to control *Cx*. *pipiens* and no substantial changes have been observed in the land use of the area and in the human population size. The biotype present in the eastern Piedmont area has not been definitively identified. However, given the relatively infrequent bites to humans in the considered area, a previous study has suggested *Cx*. *pipiens pipiens*—which is predominantly bird-feeding—as the more likely biotype [17]. It is possible that human exposure to mosquito bites may be lower in more agricultural areas. However, a recent study conducted in a region of Northern Italy showed that *Cx*. *pipiens* prefer to take blood meals from avian hosts both in rural and urban areas [35]. For a more detailed description of the study area and the trapping conditions, see [16, 17]. Data used for the proposed analysis are fully available in S1 Table.

### Modelling mosquito dynamics

The model for the dynamics of the abundance of the vector in seven life stages of *Cx*. *pipiens*, namely eggs (*E*), 4 larval instars (*L*_*1*_, *L*_*2*_, *L*_*3*_, *L*_*4*_), pupae (*P*) and non-diapausing female adults (*A*), is based on the following system of equations:

$$M=\{\begin{array}{l} E'|=\frac{n_{E}}{d_{A}}A-\left(\mu_{E}+\tau_{E}\right)E\\ L{'}_{1}|=\tau_{E}E-\left(\tau_{L_{1}}+\mu_{L_{1}}\left(1+\frac{L_{1}+L_{2}+L_{3}+L_{4}}{K}\right)\right)L_{1}\\ L{'}_{2}|=\tau_{L_{1}}L_{1}-\left(\tau_{L_{2}}+\mu_{L_{2}}\left(1+\frac{L_{1}+L_{2}+L_{3}+L_{4}}{K}\right)\right)L_{2}\\ L{'}_{3}|=\tau_{L_{2}}L_{2}-\left(\tau_{L_{3}}+\mu_{L_{3}}\left(1+\frac{L_{1}+L_{2}+L_{3}+L_{4}}{K}\right)\right)L_{3}\\ L{'}_{4}|=\tau_{L_{3}}L_{3}-\left(\tau_{L_{4}}+\mu_{L_{4}}\left(1+\frac{L_{1}+L_{2}+L_{3}+L_{4}}{K}\right)\right)L_{4}\\ P'|=\tau_{L_{4}}L_{4}-\left(\tau_{P}+\mu_{P}\right)P\\ A'|=\frac{1}{2}\tau_{P}\left(1-p\right)P-\beta \mu_{A}A-\chi_{C}\alpha A\\ C'|=\chi_{C}\alpha A \end{array}$$

where $\tau_{E},\tau_{L_{1}},\tau_{L_{2}},\tau_{L_{3}},\tau_{L_{4}},\tau_{P}$ are the temperature dependent developmental rates driving the transitions of vectors across the different life stages considered; $\mu_{E},\mu_{L_{1}},\mu_{L_{2}},\mu_{L_{3}},\mu_{L_{4}},\mu_{P},\mu_{A}$ are the temperature dependent death rates associated with the different stages; *n*_*E*_ is the number of eggs laid in one oviposition; *d*_*A*_ is the length of the gonotrophic cycle; *K* is the density-dependent scaling factor driving the carrying capacity for the larval stages; *p* is the probability (depending on daylight duration) that a fully developed pupa becomes a diapausing adult; β gauges the possible increase in adult mortality rate due to wild conditions with respect to lab conditions; α is the capture rate; *χ*_*C*_ is a function of the time defined equal to 1 when the trap is open and 0 otherwise; *C* represents the cumulative number of captured female adult mosquitoes. Since only female adult mosquitoes are explicitly considered in the model, the term 1/2 in the equation for the adults accounts for the sex ratio [36]. Note, moreover, that diapausing females do not take blood meals before overwintering [21] and they cannot be captured with the considered traps. For this reason, only non-diapausing female adults are considered in the model.

Daily mean temperature and precipitation records for the period and study area considered were obtained from ARPA Piedmont [37]. Daylight durations for the centroid of the study region during the considered period were obtained from the US Naval Observatory [38].

We actually adopted a discrete–time stochastic version of model *M*, with time–step Δ*t* = 1 day, in order to account for the stochastic nature of the processes. Precisely, the model is a Markov chain whose states represent the number (an integer) of individuals in all developmental stages, and whose transition probabilities are built according to binomial distributions whose means are obtained from the rate in system *M*. Details are specified in S1 Text. The seasonal dynamics of the mosquito population is simulated for 12 years, from April 1 (corresponding to approximately one month before the first capture session) to October 1. Since, to the best of our knowledge, no data are available on the overwintering of *Cx*. *pipiens*, we simulate each year *y* separately by initializing the system with *A*_0_(*y*) > 0 non-diapausing adults.

### Model calibration

Mortality and developmental rates across different vector life stages have been modeled as a function of temperature following the approach already proposed in [29] on the basis of data collected in [19, 39]. Specifically, we modeled the developmental period and the mortality rate associated with different vector stages at each temperature by fitting a suitable set of functions of the temperature *T*—comprising exponential and parabolic functions—to durations and rates measured at different specific temperatures through laboratory experiments [19, 39]. For the egg developmental rate, we used the same function proposed in [32]. The same technique was used to estimate the probability *p* for a developed pupa to become a diapausing adult as a function of daylight duration using the data presented in [22]. The uncertainty of parameters’ estimates was obtained by using a bootstrap procedure similar to that used in [29, 40]. More details on the technique employed are presented in S1 Text.

To the best of our knowledge, data on adult mortality at different temperature are not available for *Cx*. *pipiens*. Therefore, the mortality rate of adult female mosquitoes has been taken as the function of temperature suggested in [18], also allowing for an increase in adult mortality rate in the wild relatively to lab conditions. The average number of laid eggs *n*_*E*_ per oviposition and the duration of the gonotrophic cycle *d*_*A*_ in our simulations were chosen uniformly in the intervals [150,240] and [2,8] days respectively, according to results presented in [41,42].

Free model parameters to be estimated are the capture rate α, the increase of adult death rate in the wild β, the density-dependent factor *K*, and the number of initial adults *A*_*0*_. More specifically, we assumed α and β to be equal among all years considered, while the value of *K* and *A*_*0*_ could be year-specific.

Model predictions for the dynamics of mosquito population during a specific season depend on the free parameters θ = (α, β, *K*, *A*_0_) but are also influenced by the intrinsic stochasticity of simulations and by the uncertainty on parameters defining the transition rates used in the model (e.g. the developmental and mortality rates for different mosquito life-stages). By denoting the latter set as *ω* we define as *λ*_{*m*,*y*}_(*θ*, *ω*) the number of captures at month *m* and year *y* predicted by the model with parameters θ and *ω*.

In order to estimate the free parameters by taking into account both the stochasticity of the process and the uncertainty on parameter estimates defined by *ω*, for each year *y*, we define the expected number of captures at month *m* associated with θ, denoted hereafter by ${\tilde{\lambda }}_{\left\{m,y\right\}}(\theta )$, as the *λ*_{*m*,*y*}_(*θ*, *ω*) corresponding to the simulation producing the median cumulative number of yearly captures among the simulations obtained by employing the same parameter set θ and varying *ω*.

The posterior distributions of the free parameters θ were explored by Markov chain Monte Carlo (MCMC) sampling applied to the likelihood of observing the monthly number of trapped adults, averaged among the 24 considered sites. Assuming that for each month the number of observed trapped adult mosquitoes follows a Poisson distribution with mean obtained from the model, the likelihood of the observed data over the twelve simulated years has been defined as

$$L=\overset{2011}{\underset{y=2000}{\prod }}\overset{5}{\underset{m=1}{\prod }}e^{\left\{-{\tilde{\lambda }}_{\left\{m,y\right\}}\left(\theta \right)\;)\right\}}\frac{{\tilde{\lambda }}_{\left\{m,y\right\}}\left(\theta \right){\;}^{n_{\left\{m,y\right\}}}}{n_{\left\{m,y\right\}}!}$$

where *y* runs over the different considered years, *m* runs over months, n_{m,y}_ is the observed average number of trapped adults over the 24 sites at month *m* and year *y* as reported in [16, 17] and ${\tilde{\lambda }}_{\left\{m,y\right\}}(\theta )$ is the predicted number of captures at month *m* and year *y* simulated by the model with parameters θ = (α, β, *K(y)*, *A*_0_(*y*)).

The posterior distribution of θ was obtained by using random-walk Metropolis-Hastings sampling approach [43] and normal jump distributions. A total of 100,000 iterations were performed and a burn-in period of 5,000 steps was chosen. Convergence was checked by considering chains associated with different starting points in the parameter space and by visual inspection on the trace plots of chains.

Model predictions associated with the estimated posterior distributions of model parameters for the different seasons (from 2000 to 2011) were analyzed in terms of *i*) the weekly number of *Cx*. *pipiens* captured during the twenty-week survey period; *ii*) the total number of captured mosquitoes at the end of each year; *iii*) the highest weekly capture during each year; *iv*) the week at which the highest capture was observed; *v*) the start and the end of the mosquito season, defined as in [17] to be the weeks by which respectively 5% and 95% of the cumulative captures in the simulated season occurred; for clarity, from now on, we will denote these values by onset and offset; *vi*) the season length, defined as the number of weeks between the onset and the offset of the season [17]. The uncertainty surrounding model predictions is generated by both the variability of the estimated posterior distribution of free model parameters and the intrinsic stochasticity characterizing model simulation.

Finally, we applied the model to assess the influence of the temperature on the population dynamics. To this aim, we simulated each year *y* with 10 different temperature patterns *T(y*,*t)* ranging from $\overset{-}{T(y,t)}-2.5{}^{\circ}\;$ to $\overset{-}{T(y,t)}+2.5{}^{\circ}$, where $\overset{-}{T(y,t)}\;$ is the observed temporal pattern of temperature associated with year *y*. Following a similar approach, we investigated the role played by the larval carrying capacity by simulating each year *y* with different density-dependent factors *K*, ranging from $0.5\cdot \overset{-}{K(y)}\;$ to $1.5\cdot \overset{-}{K(y)}$, where $\overset{-}{K(y)}\;$ is the estimated density-dependent factor for year *y*.

## Results and Discussion

**Fig 1:**
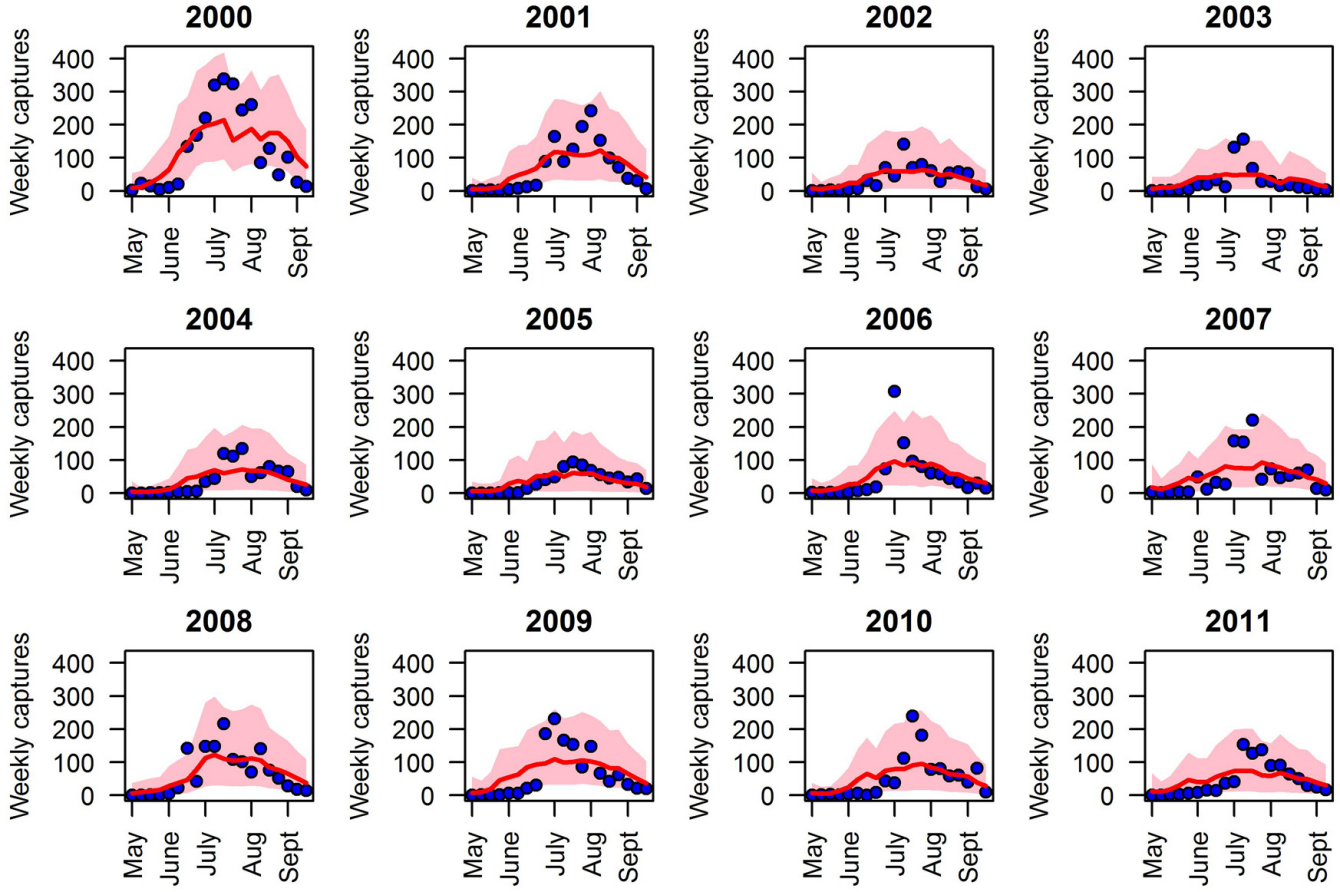
Model fit. Average number of weekly captured *Cx*. *pipiens* during the twenty-week survey period observed in Piedmont region from 2000 to 2011 (blue points) and predicted by model simulation based on the estimated posterior distribution of free parameters (median in red, pink region defines 2.5–97.5% quantile predictions).

The proposed model can well reproduce the number of weekly captures of adult mosquitoes reported between May and September for all the twelve years of observation (2000–2011). In particular, more than 90% of the weekly trap records lie within the 2.5–97.5% quantile of model predictions. The model shows the ability of reproducing both the strong seasonality characterizing the adult population dynamics within different years and the high heterogeneity observed among different seasons in terms of mosquito density (see Fig 1).

**Fig 2:**
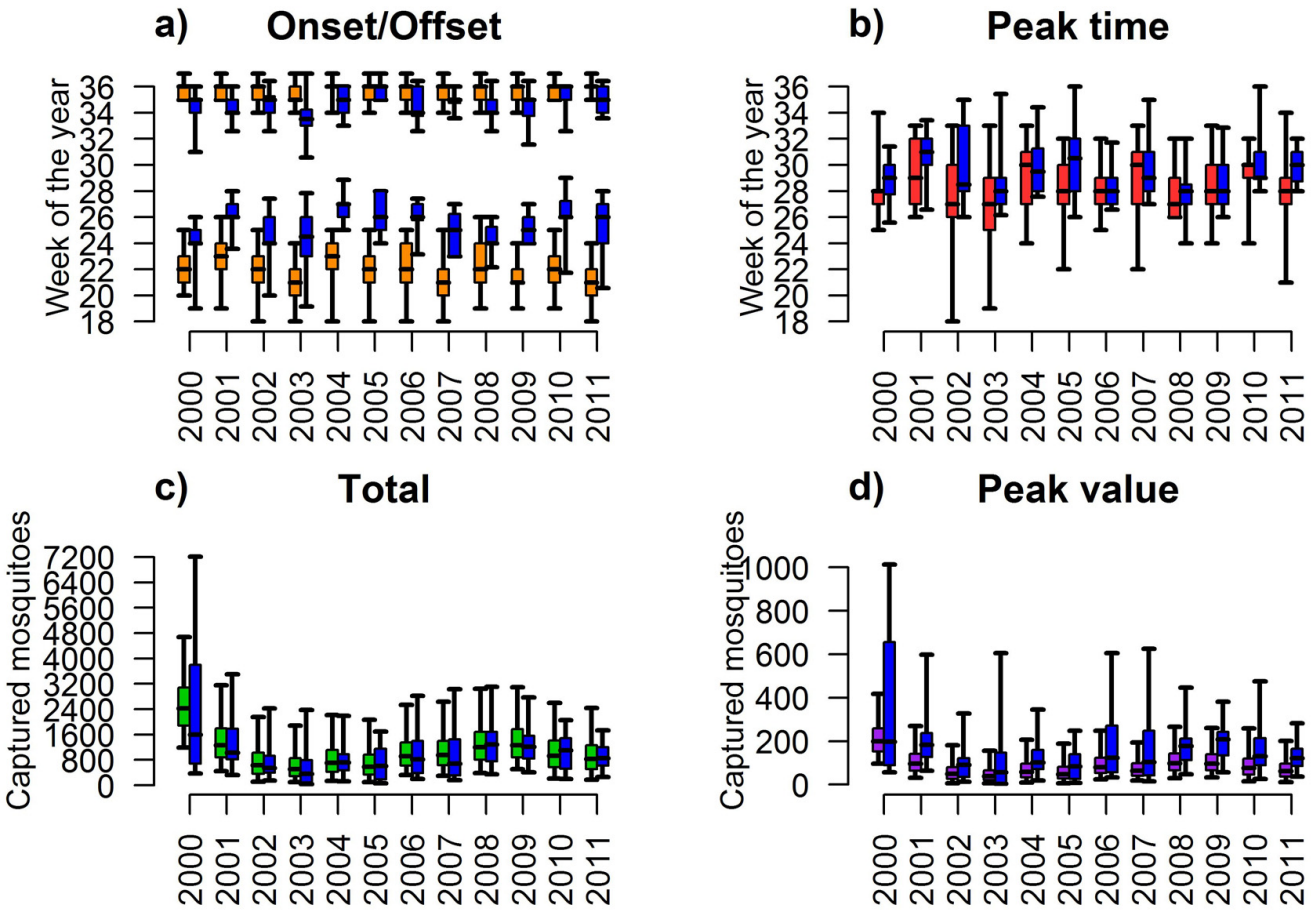
Annual synthetic indexes. Boxplot (2.5%, 25%, 75% and 97.5% quantile and median) of predicted onset (lower orange bars in panel a) and offset (higher orange bars in panel a), defined as the week of the year when the 5% and the 95% of the cumulative captures are reached respectively; week of the year associated with peak (highest) capture (red bars in panel b); total annual captures, i.e. the sum of the 20 weekly captures (green bars in panel c); peak capture, i.e. maximum number of trapped adults in a single capture session (purple bars in panel d). Blue boxplots represent the distributions of the observed site-specific values. Distributions of the observed peak capture were obtained by computing the maximum of 3-point moving average of weekly captures.

In agreement with collected data (see Fig 2c), our results show that the average cumulative number of trapped adults can substantially change between seasons, ranging from 510 (2.5–97.5% quantile predictions: 100–1887) in 2003 to 2425 (2.5–97.5% quantile predictions: 1194–4677) in 2000.

The highest capture is predicted to occur, on average, between the 27^th^ and 31^st^ week of the year (corresponding to the month of July) in good agreement with observed values (see Fig 2b). On the opposite, the predictions on the maximum number of trapped adults in a single capture session during the entire season are extremely variable among different simulations and do not accurately reproduce observed values. This field measure is highly sensitive and reflects stochastic variations driven by site-specific factors such as rain and wind condition of the day. Indeed, strong wind and rainfalls might alter *Cx*. *pipiens* dispersal and host-seeking behavior, possibly reducing the probability of being captured. In fact, data collected show that captures of two consecutive trapping sessions can be remarkably different (with differences sometimes of an order of magnitude). In order to smooth the inherent variability in captures, we computed, for each trap, the 3-point moving average of weekly captures. The distribution of the maxima of moving averages, for each year, is shown in Fig 2d; it can be seen that the variability in model predictions is consistent (though a bit lower) with the observed variability among traps. In addition, years characterized by higher maximum number of trapped adults within a single capture are associated with higher peaks in model predictions.

The 2.5–97.5% quantile of the predicted offsets are between the 34^th^ and 37^th^ week (mid-August—mid-September) in each year, like the observed captures (see Fig 2a). The predicted onsets are on the average between the 21^st^ and the 23^rd^ week (end of May–beginning of June), a few weeks earlier than what observed (median values between 24^th^-27^th^ week, June).

In [17] the authors found that the starting time of the mosquito season (onset) was negatively correlated with the average temperature of weeks 8–19 (i.e., higher temperatures hasten the onset), and found that season length was positively correlated with mean temperature of weeks 16–27. Our analysis confirms such results suggesting that the median predicted onset, which defines the starting time of the season, is negatively correlated (y = 27.81−0.52⋅x, p-value<0.01) with the average temperature recorded between mid-February and the beginning of May, which ranges from 9.6°C in 2004 to 13.4°C in 2007. This is in line with the observed faster development of immature stages associated with higher temperatures.

Furthermore, the median predicted season length is positively correlated (y = −2.00 + 0.80⋅x, p-value<0.01) with the average temperature recorded between mid-April and the end of June, which varies from 18.5°C in 2004 to 21.5°C in 2003.

The model accounts for the observed heterogeneous dynamics of the mosquito population among different seasons thanks to the explicit inclusion of two seasonal factors. The first one is the dependence of developmental and mortality rates of different mosquito stages on temperature. The second one is represented by the assumption of a year specific density-dependent factor for larval stages, which may reflect possible differences in the availability of breeding sites in different years.

**Fig 3:**
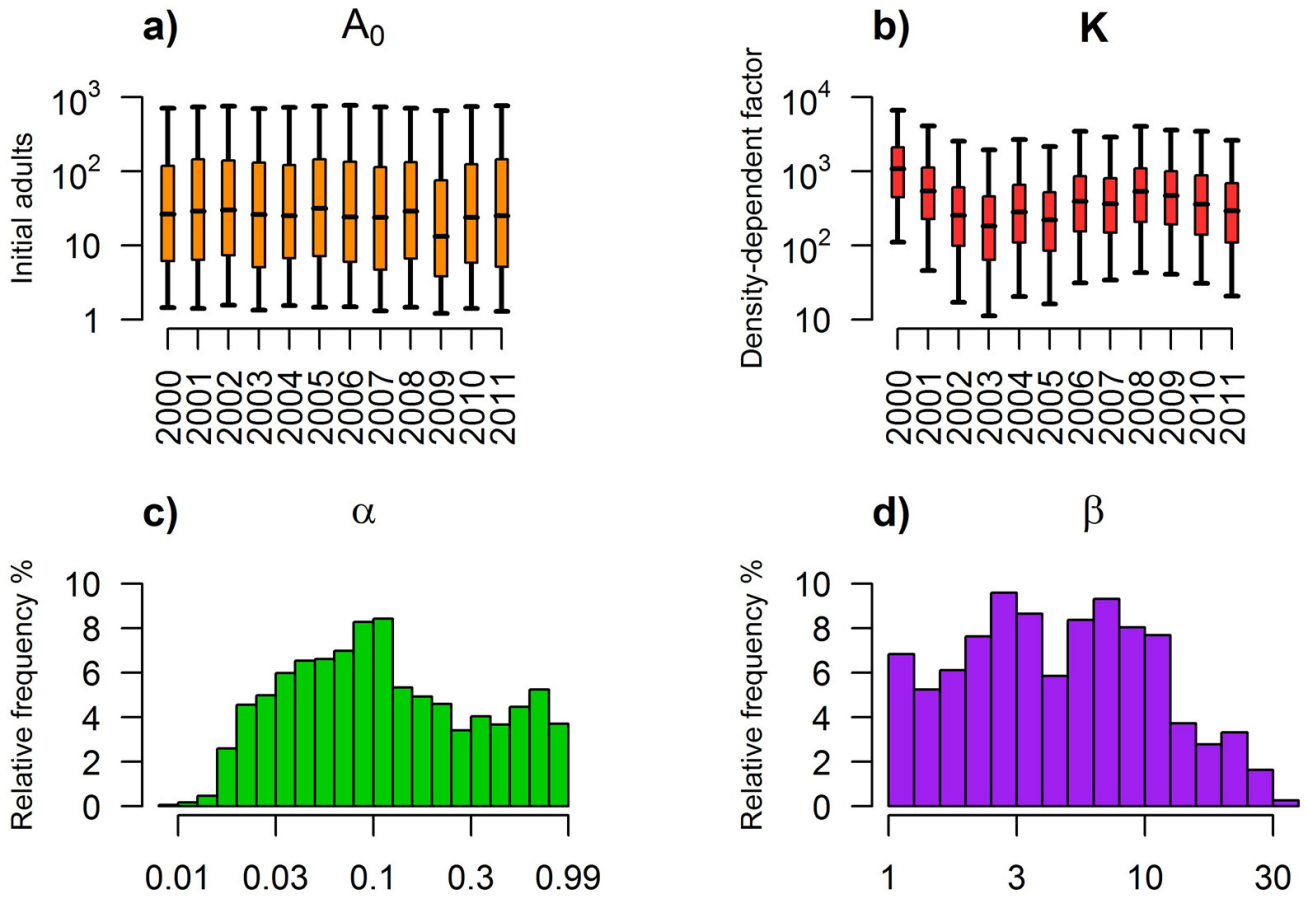
Estimated parameters. Boxplot (2.5%, 25%, 75% and 97.5% quantile and median) of posterior distributions of parameters *A*_*0*_ (panel a) and *K* (panel b) estimated in different years. Histograms of relative frequencies for posterior distributions of parameters α (panel c) and β (panel d).

The estimated posterior distribution of the initial number of adults (namely A_0_) spans a wide range, between approximately 1 and 1,000 in each season (see Fig 3a), with negligible differences among different years.

Conversely, estimated posterior distributions of the density-dependent factor are remarkably different among years (see Fig 3b). A recent study [17] carried out on the same dataset considered in this work has shown that *Cx*. *pipiens* population size in different years is positively correlated with the number of days of precipitation in the first three months of the year. Following the same approach presented in [17], we explored possible correlations between the estimated density-dependent factors and the number of rainy days among different temporal windows. We considered 22 temporal widows built by grouping periods of 12 consecutive weeks, starting from the first week of the year (weeks 1–12) and ending with weeks 22–33. For each window, number of days of precipitation was summed. We found that the median value of the estimated density-dependent factor is positively correlated (y = -380.54+ 32.14⋅x, p-value<0.01) with the number of rainy days in weeks 13–24 (end of March—mid-June), which encompass partially the first half of the simulated period. Therefore, although the model does not take precipitation explicitly into account, our analysis highlights its likely influence on mosquito population dynamics. This positive correlation is biologically reasonable as more rain can create more breeding sites and therefore increase the carrying capacity of larval stages, which is proportional to the density-dependent factor.

The capture rate α is estimated to be on average 11.35% (10% median, see Fig 3c) in good agreement with values published in [44], where it was estimated to be 10.8% through a field experiment carried out using bird-baited traps placed outdoors in an open lawn area. However, it is worth noting that this experiment was carried out in a setting different from our study area, using different traps. Furthermore, the posterior distribution we obtained for α is very wide, and thus does not give strong support to any specific estimate.

The estimated posterior distribution of β, the increase in adult mortality rate in the wild relatively to lab conditions, is also very wide (95% CI 1.09,23.98, see Fig 3d) with an estimated average of 4.61 (4.52 median). This result is in good agreement to what has been observed in [45] for *Aedes albopictus*, for which adult survival is four times lower in the wild relatively to the survival observed under laboratory conditions.

Undoubtedly, independent estimates on a subset of our free parameters would allow providing more robust estimates of these specific biological quantities. However, the MCMC approach represents a suitable statistical technique to handle uncertainties about parameters, as it takes into account all possible parameters’ configurations compliant with patterns observed in the data. Simulations were run also by assuming seasonal dependent α and β. The two different modeling assumptions result in qualitatively similar predictions about the abundance of the mosquito among different years (see S1 Text). These results strongly suggest that the more parsimonious model with seasonal independent α and β should be preferred as associated with a lower value of the Deviance Information Criterion (DIC) [46].

**Fig 4:**
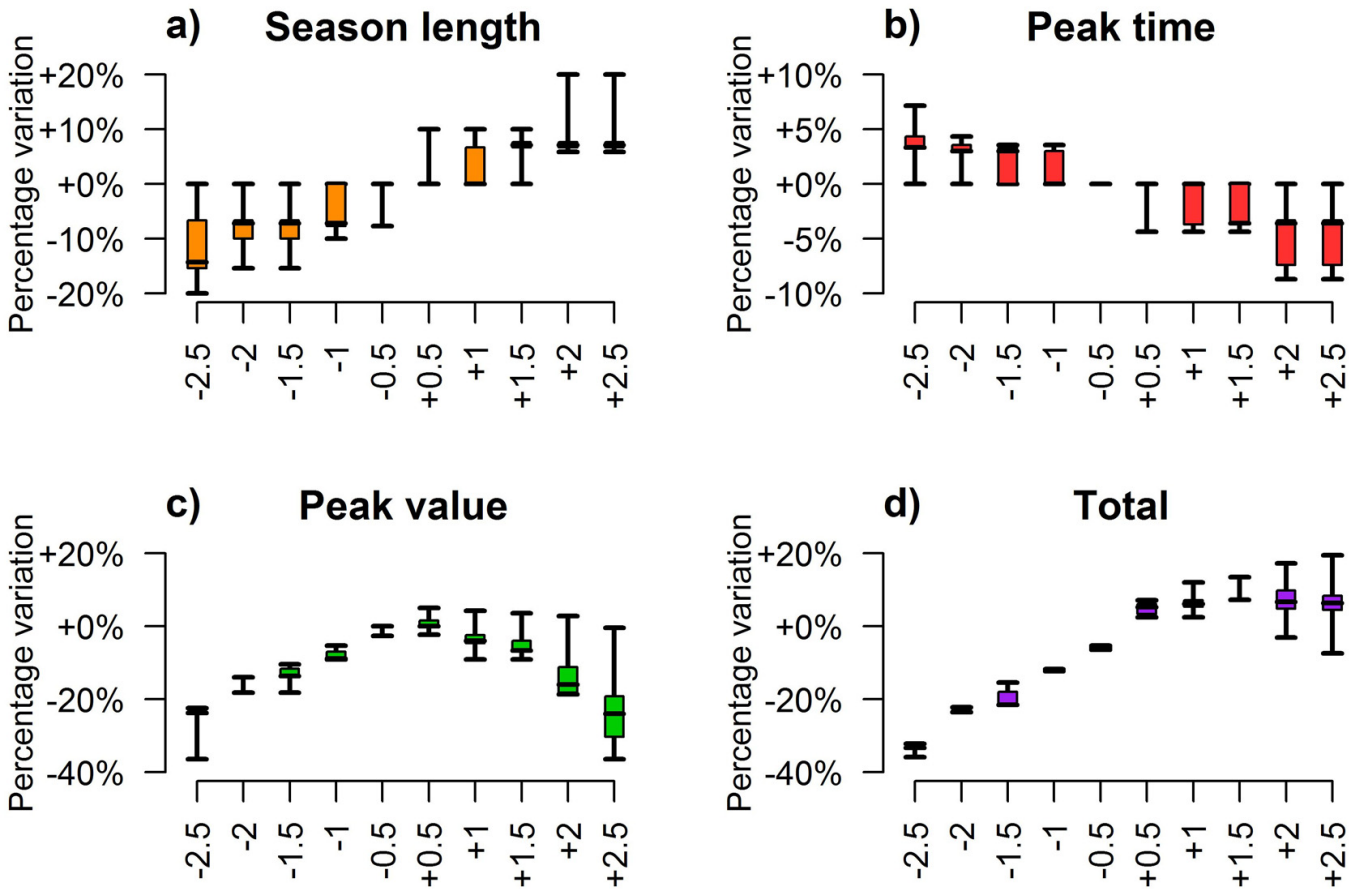
Effect of temperature variations on *Cx*. *pipiens*. Boxplots (2.5%, 25%, 75% and 97.5% quantile and median) of predicted annual synthetic indexes associated with different temperature inputs (x-axis, from -2.5°C to +2.5°C with respect to actual records). Panel (a) shows the effect on the duration of the breeding season, defined as the difference between the week of the year when the 95% and the 5% of the cumulative captures are reached; panels (b) and (c) show respectively the effect on the timing and the value of the peak capture; panel (d) shows the effect on the total annual captures.

Temperature plays a crucial role in shaping the population dynamics of *Cx*. *pipiens*. As already suggested by the statistical correlations presented above, higher temperatures can both hasten the occurrence of high adult densities (see Fig 4b) and lengthen the breeding season (see Fig 4a). On the other hand, either too high or too low temperatures during the season might be responsible of a noticeable decrease in peak mosquito abundance (see Fig 4c) as a consequence of the balance between two opposite phenomena; high temperatures increase mosquito mortality rates (especially in adults) while low temperatures can strongly reduce the developmental rates of mosquito immature stages. Our results suggest that a reduction of the temperature of 1.5°C decreases both the highest mosquito density during the season and the cumulative number of captured mosquitoes of about 20%, while in the extreme case of a decrease of 2.5°C a reduction of 40% of the total abundance and peak values is expected (see Fig 4d). This confirms that the inability of immature stages to cope with low temperature is a critical factor in shaping *Cx*. *pipiens* habitat suitability.

Hotter seasons might also reduce the maximal abundance of adult mosquitoes (about -25% for the +2.5°C scenario) but produce only negligible effects on the overall number of captured adults during the whole season. This apparent contradiction can be explained by the observation that higher temperatures increase mosquito populations during spring and decrease them during summer (see S1 Text).

**Fig 5:**
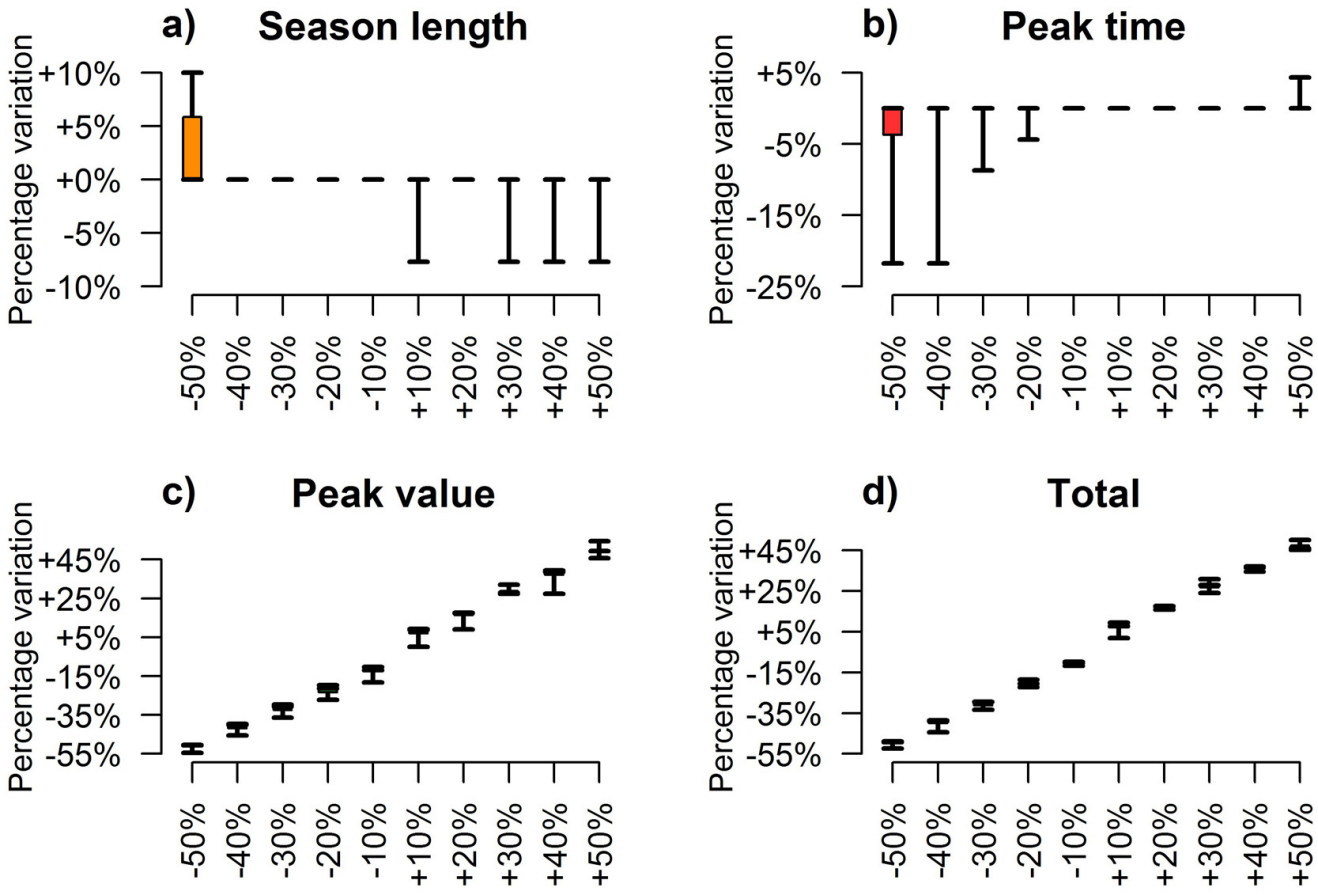
Effects of density-dependent factor variations on *Cx*. *pipiens*. Boxplots (2.5%, 25%, 75% and 97.5% quantile and median) of predicted annual synthetic indexes associated with different values of *K* (x-axis, from -50% to +50% with respect to fitted values). Panel (a) shows the effect on the duration of the breeding season; panels (b) and (c) show respectively the effect on the timing and the value of the peak capture; panel (d) shows the effect on the total annual captures.

On the other hand, changes in the larval carrying capacity produce proportional effects on mosquito abundance during the whole breeding season. For instance, our analysis shows that a 30% reduction of the density-dependent factor *K* causes a decrease of about the same percentage on both the highest capture and the cumulative number of captured adult mosquitoes (see Fig 5c and 5d). Lower values of the larval carrying capacity prevent the development of a large number of larvae into pupae and, in turn, into adults. Consequently, under favorable conditions, an increase of this parameter increases the population size. However, the length of the breeding season and the time of the highest capture are not significantly influenced by the magnitude of larval carrying capacity. Indeed, the occurrence of favorable conditions, such as the increase of the developmental rates of aquatic stages into adults, is mainly driven by temperature. These results suggest that the carrying capacity, which correlates with the abundance of spring precipitations and is possibly linked to the availability of mosquito breeding sites, affects the reproduction number, and thus the growth rate, of the population but it does not influence the developmental and the mortality rates at the beginning and at the end of the season, which are the main determinants of season length.

## Conclusions

In this paper, we investigated which are the main drivers of the observed high heterogeneity characterizing the *Cx*. *pipiens* population among different seasons in Northwestern Italy. We found that inter-seasonal variability is determined by two main drivers: i) differences in larval carrying capacities, which in turn might depend on the cumulative number of rainy days from end of March to mid-June, potentially correlated to the availability of breeding sites, and ii) differences in average temperatures, which affect both developmental and survival rates.

Overall, this work provides useful indications about the dynamics of *Cx*. *pipiens* during a typical breeding season. Our results suggest that variations in the number of rainy days and temperature, like those observed in the study period, may give rise to substantially different seasonal mosquito abundances and provide interesting insights on how possible climatic changes could affect the future density of this vector in Piedmont and in similar areas.

The data also exhibit a large degree of spatial heterogeneity, as trap captures vary in abundance and temporal patterns. Investigating these patterns would require detailed information on habitat utilization and related mosquito movement, which are not available and are beyond the scope of the present work. Instead, data coming from different traps were aggregated in order to strengthen the investigation of seasonal heterogeneity, by reducing the influence of climatic condition characterizing single specific days.

In this work, *Cx*. *pipiens* population dynamics has been modeled on the basis of the empirical relations found in laboratory experiments between demographic and developmental rates of the various life stages (eggs, larvae, pupae, female adults) on temperature and, as far as diapause is concerned, photoperiod. Use of statistical methods on population data have allowed us to use the model with field data, elucidating the role of density-dependence. Availability of data on survival and fertility rates in the wild—where for instance *Cx*. *pipiens* adults are expected to seek refuge from heat in summer and from cold in winter—could allow for refinements of the model and for using it beyond a single season.

## Supporting Information

S1 Table Dataset used for model calibration: weekly trapped adult mosquitoes for 12 consecutive years (2000–2011). (XLSX)

S1 Text Supporting text containing methodological details and additional results. (PDF)
