# Supplementary material for: The Role of Climatic and Density Dependent Factors in Shaping Mosquito Population Dynamics: The Case of Culex pipiens in Northwestern Italy
Source: PLoS One. 2016 Apr 22;11(4):e0154018. doi: 10.1371/journal.pone.0154018 (PMC4841511; doi:10.1371/journal.pone.0154018)
Supplement: S1 Text — (PDF) [file pone.0154018.s002.pdf]

The Role of Climatic and Density Dependent Factors in Shaping Mosquito Population Dynamics: the Case of *Culex Pipiens* in Northwestern Italy

Giovanni Marini[1,2], Piero Poletti[3,4], Mario Giacobini[5], Andrea Pugliese[1], Stefano Merler[3], Roberto Rosà[2]

[1]Department of Mathematics, Trento University, Trento, Italy;

[2]Department of Biodiversity and Molecular Ecology, Fondazione Edmund Mach, San Michele all'Adige, Italy;

[3]Bruno Kessler Foundation, Trento, Italy;

[4]Dondena Centre for Research on Social Dynamics and Public Policy, Department of Policy Analysis and Public Management, Universitá Commerciale L. Bocconi, Milan, Italy;

[5]Department of Veterinary Sciences, University of Torino, Torino, Italy;

Supporting Text S1

Materials and methods

Model calibration

In this manuscript, we introduce a model to investigate the vital dynamics of *Culex pipiens* during a typical breeding season by considering temperature-dependent mortality and developmental rates both for immature and mature/adult stages of mosquito and by assuming that the diapause rate depends on daylight duration.

The lengths of the developmental periods associated to different mosquito life stages (i.e. for any $s \in S = \{L_1, L_2, L_3, L_4, P\}$) at different temperatures were calibrated according the following procedure. Given the length of developmental period L_s for temperatures $T \in \mathcal{T} = \{7°C, 10°C, 15°C, 20°C, 25°C, 30°C, 33°C\}$ as observed in [1], we assume that $L_s(T) = f_s(T;\omega) + \varepsilon_T$ where $f_s(T;\omega)$ is a parametric function of the temperature T (ω indicates the set of free model parameters defining f) in a suitable set of functions, comprising exponential, parabolic and logistic functions, and ε_T is a random sample of a 0 mean normal distribution with unknown variance σ^2. For each considered life stage, we calibrate the function $f_s(T;\omega)$ by minimizing the square error φ between predicted and observed length of the period which is defined as $\varphi = \sum_{T\in\mathcal{T}} \left(L_s(T) - f_s(T;\omega)\right)^2$.

Uncertainty of estimated parameters (i.e., ω) was computed following a bootstrap procedure similar to the one adopted in [2, 3]. In particular, we simulated 100 different $\{L_s(T)\}_{T\in\mathcal{T}}$ by adding an error sampled from a normal distributed $N(0,\sigma^2)$ to the best interpolation $f_s(T;\widetilde{\omega})$ where the variance σ^2 was taken as the average of the estimated residuals associated to the best interpolation of the model i.e., the average of the quadratic differences $\left(L_s(T) - f_s(T;\widetilde{\omega})\right)^2$. Finally, for each simulated $\{L_s(T)\}_{T\in\mathcal{T}}$ we repeated the optimization procedure described above. Obtained estimates of $l_s(T;\omega)$ were used to compute the rate of development as $\tau_s(T;\omega) = 1/l_s(T;\omega)$.

The same technique was applied to estimate the probability *p* for a fully developed pupa to become a

diapausing adult as function of the daylight duration using the data presented in [4] and to estimate the mortality rates of all immature stages as functions of temperature.

However, since mortality data for different mosquito life-stages were available as survival probabilities, an additional step was required to estimate the associated mortality rates. The survival probability for different mosquito life-stages observed in [1, 5] was obtained in lab conditions by following a cohort of *n* individuals until all of them would either die or develop in the subsequent life stage.

In our analysis, we estimate the mortality rates associated to eggs, different larval instars and pupae by maximizing the likelihood of observing the number of surviving individuals *k* obtained in the lab experiments at fixed temperatures, given the initial number of individuals *n* and a known developmental rate for each temperature and each mosquito life-stage.

For instance, specializing model *M* to the case of a cohort of pupae kept at a fixed temperature $\bar{T}$ and starting at time 0 with the initial value $n(\bar{T})$, one sees that, following the approach described in [6], the probability that a pupa will eventually develop into an adult (instead of dying) is $p(\bar{T}) = \frac{\tau_P(\bar{T})}{\mu_P(\bar{T})+\tau_P(\bar{T})}$, so that the number of pupae developing into adults will be a binomial of parameters $n(\bar{T})$ and $p(\bar{T})$.

A simple computation shows then that the maximum likelihood estimate of $\mu_P(\bar{T})$ is

$$\mu_P(\bar{T}) = \frac{\tau_P(\bar{T})(1-p)}{p} \text{ with } p=k/n.$$

Estimates of developmental times and mortality rates at different temperatures and the diapause rate associated with different daylight durations are presented in Fig. A. Our analysis suggests that at higher temperatures, survival probabilities of all stages decrease and developmental times shorten, while longer daylight durations reduce the probability that a fully developed pupa becomes a diapausing adult. In the case of eggs and pupae, lower temperatures (i.e., below 15°C) can increase the death rate as well.

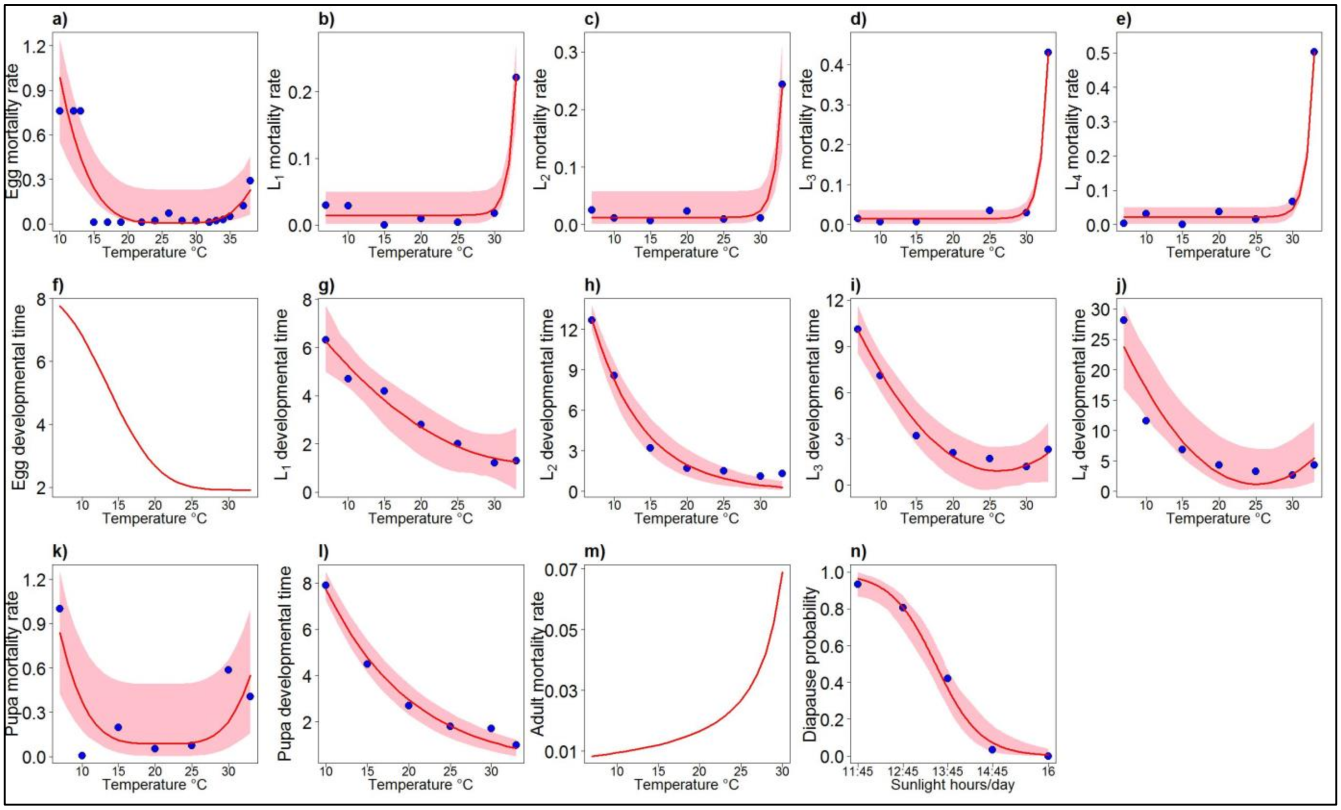

Figure A. Mortality and developmental rates at different temperatures. Panels (*a-l*): estimated mortality rates *(day^{-1})* and developmental times (in days) at different temperatures for the eggs, the larval stages and the pupal stage (i.e., *E, L_1, L_2, L_3, L_4, P*). Panel (m): adult mortality rate *(day^{-1})* modeled as function of the temperature as published in [7]. Panel (n): probability of a fully developed pupa to become a diapausing adult, modeled as function of the daylight duration. Data from experiments are shown with blue dots, red lines represent the best interpolation and pink region defines the 95% credible intervals obtained through the bootstrap procedure described in the text.

According to our model formulation, predictions of mosquito dynamics during each breeding season are driven by daylight duration (see Fig. B) and by observed seasonal temperatures (see Fig. C). The average temperature recorded from April 1st to September 30th during all the study period ranges from 19.3°C (2002) to 21.3°C (2003).

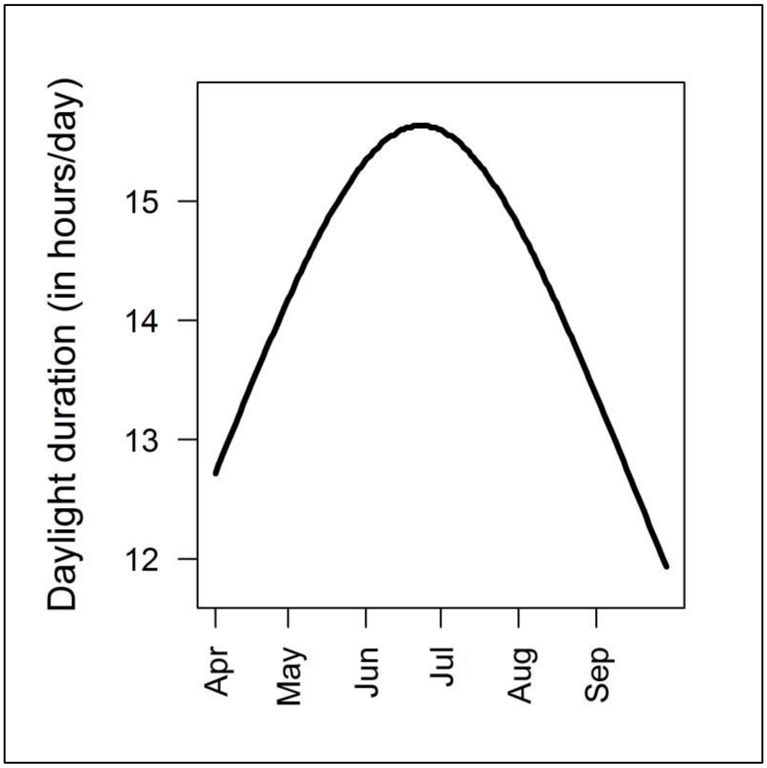

Figure B. Daylight duration during a typical breeding season. Daylight duration defined as the difference between the time of the sunset and the time of the sunrise (in hours) from April 1 to September 30 obtained from the US Naval Observatory [8].

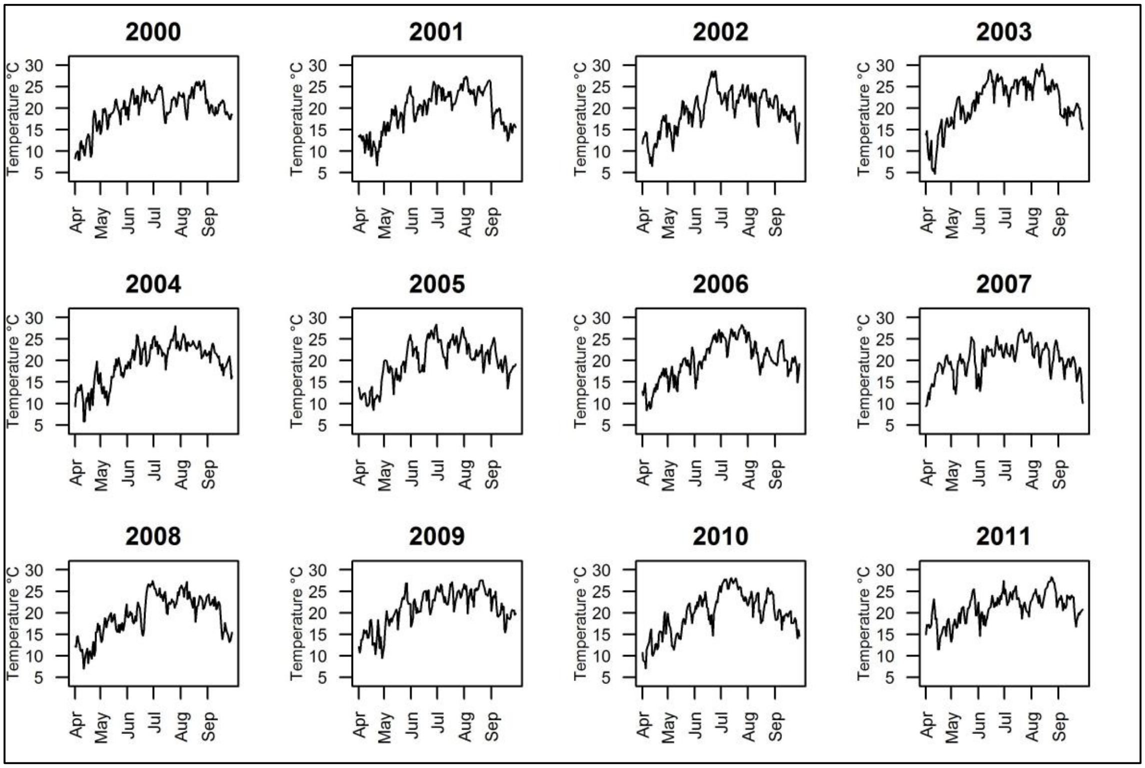

Figure C. Temperature patterns among different breeding seasons. Air mean temperatures recorded from April 1 to September 30 during the twelve years considered in the model.

Transition probabilities

Different life-stages of mosquito population are updated from time *t* to time *t+1* according to the following procedure. Given a specific population class *c* we denote the set of possible transition from the class *c* to other population classes as T where each specific transition $\tau\epsilon\mathrm{T}$ occurs at a specific rate r_τ. In our simulations at each time step *t* the number of individuals *k* leaving the class *c* is drawn from a binomial distribution $\mathcal{B}(n, \sum_{\tau\epsilon\mathrm{T}} r_\tau\,\Delta t)$, where *n* is the number of individuals in class *c* at time *t*. The number of individuals following the different transitions $\tilde{\tau}\epsilon\mathrm{T}$will then be computed from a multinomial with parameters *k* and $r_{\tilde{\tau}}/\sum_{\tau\epsilon\mathrm{T}} r_\tau$.

For instance, in case of eggs, at each time *t* eggs can either develop into larval instar at a rate τ_E or die at a rate μ_E. If *E* is the number of eggs at time *t*, first one will obtain the total number of exits, *k,* by drawing a number from a binomial distribution $\mathcal{B}(E, \mu_E\Delta t + \tau_E\Delta t)$; then the number of new larvae will be drawn from a binomial of parameters *k* and $\tau_E/(\mu_E + \tau_E)$ (correspondingly the number of dead eggs will be a binomial of parameters *k* and $\mu_E/(\mu_E + \tau_E)$).

Additional results

The proposed model is able to well reproduce the annual variations and the high heterogeneity observed in mosquito population dynamics among different breeding seasons (see Fig. D).

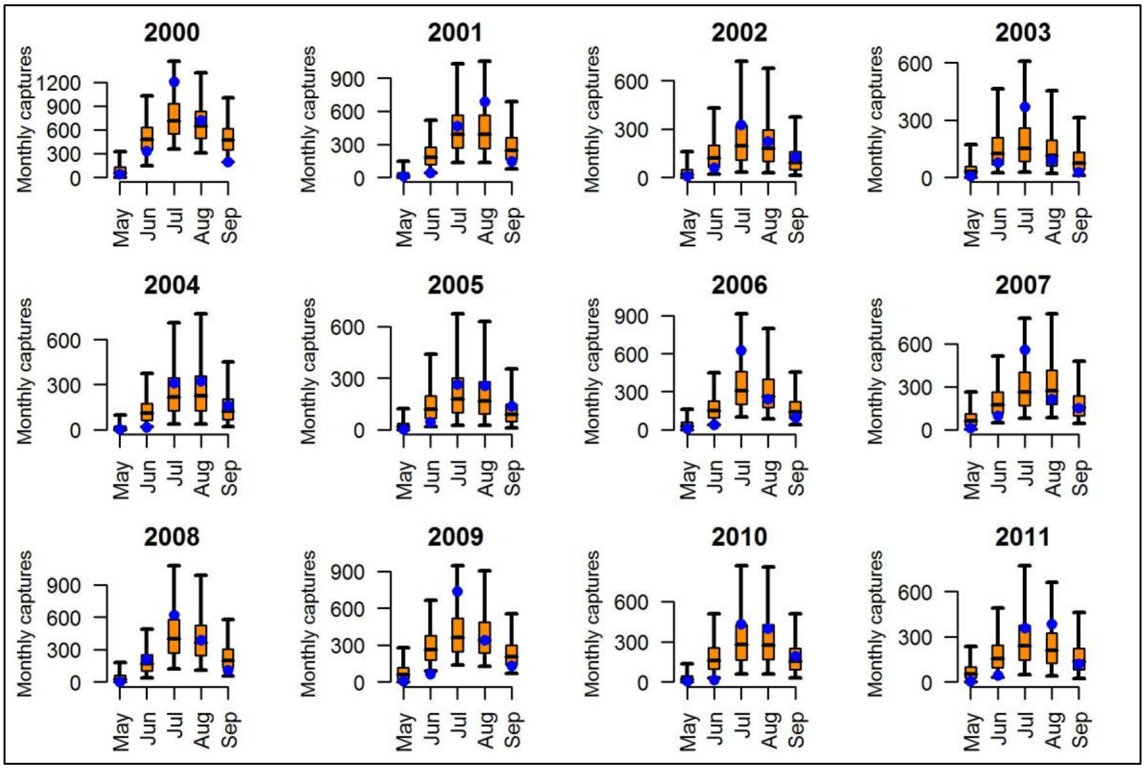

Figure D. Model fit for monthly captures. Boxplots (2.5%, 25%, 75% and 97.5% quantile and median) of fitted posterior distribution for *Culex pipiens* monthly captures. Blue dots represent the observed values.

Fig. E shows the effects of the perturbation of the observed temperature on *Culex pipiens* dynamics. In particular, for the hotter scenarios the abundance of mosquito population in spring is higher and begins to increase sooner while mosquito abundance during the summer is much lower.

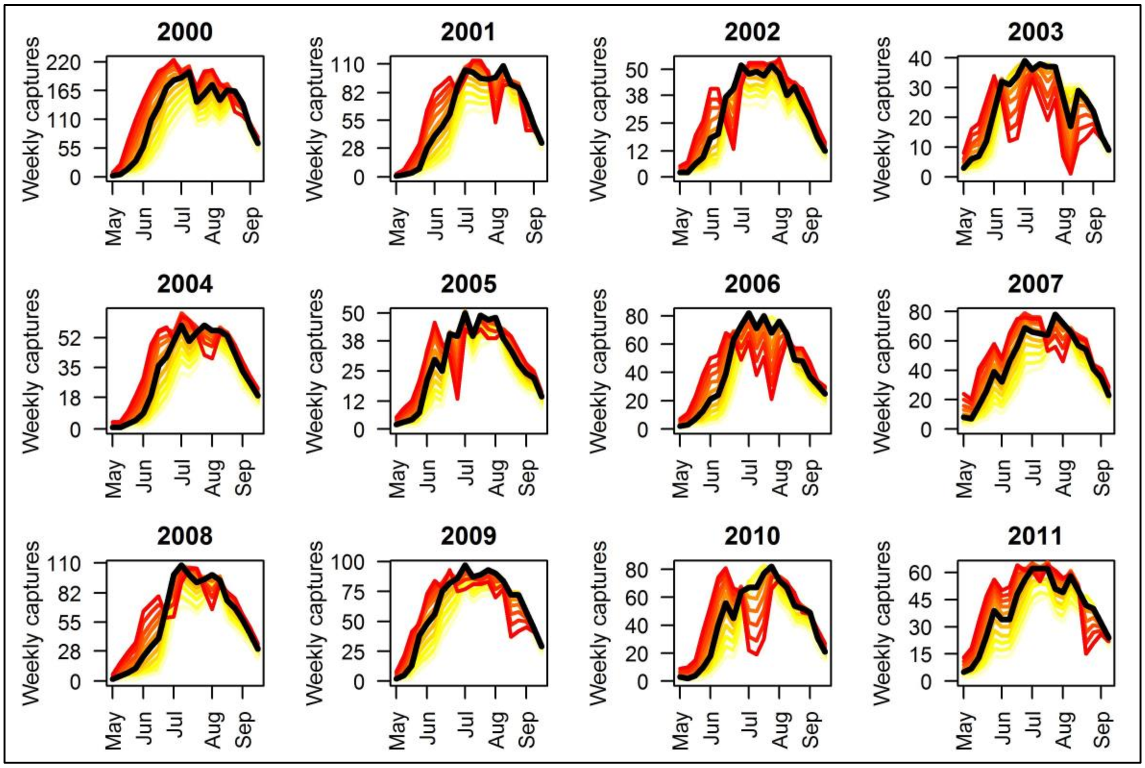

Figure E. Effect of temperature on *Culex pipiens*. Predicted captures (median values) for 10 different scenarios obtained varying daily temperature *T* from *T*-2.5°C (light yellow) to *T*+2.5°C (dark red) with a step of 0.5°C Black lines represent the median of the fit using daily temperature *T*, as shown in Fig. 1 in the main text.

The effects due to the perturbation of the density-dependent factor are shown in Fig. F. Obtained results suggest that an increase [decrease] of this parameter causes an upper [lower] shift of the entire curve.

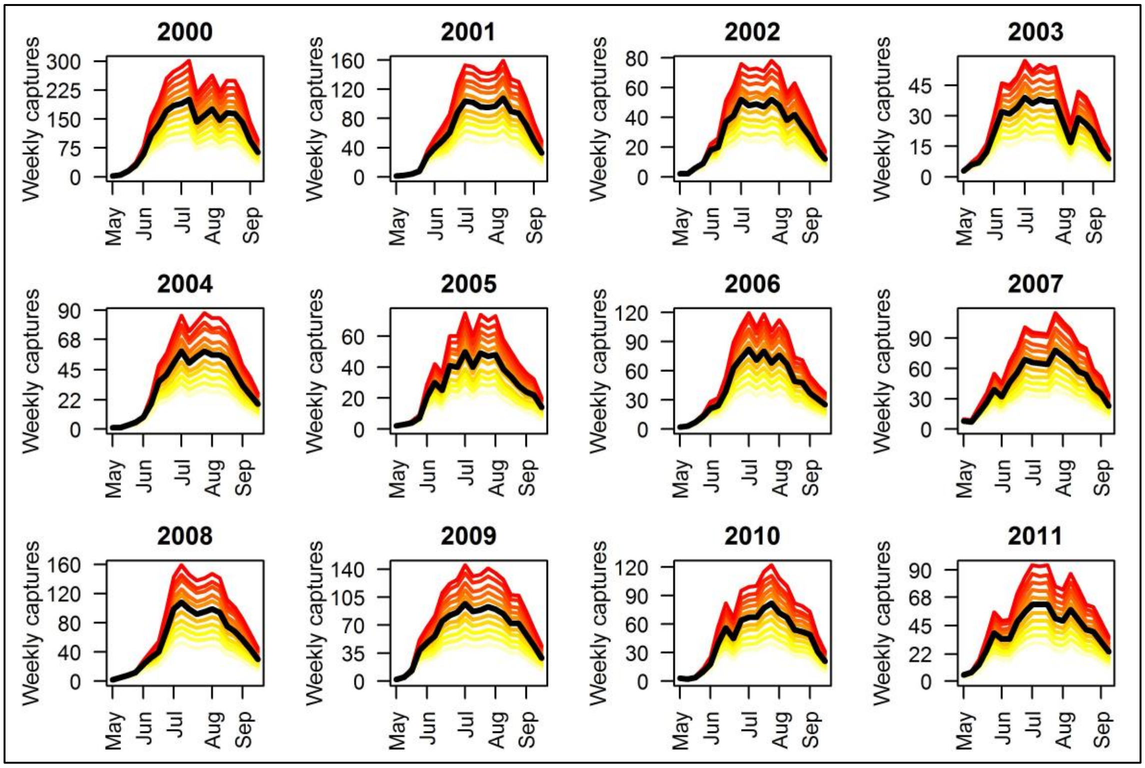

Figure F. Effect of the carrying capacity on *Culex pipiens*. Predicted captures (median values) for 10 different scenarios obtained changing the density-dependent factor *K* from $K \cdot 0.5$ (light yellow) to $K \cdot 1.5$ (dark red) with a step of $K \cdot 0.1$. Black lines represent the median of the fit, using *K,* as shown in Fig. 1 in the main text.

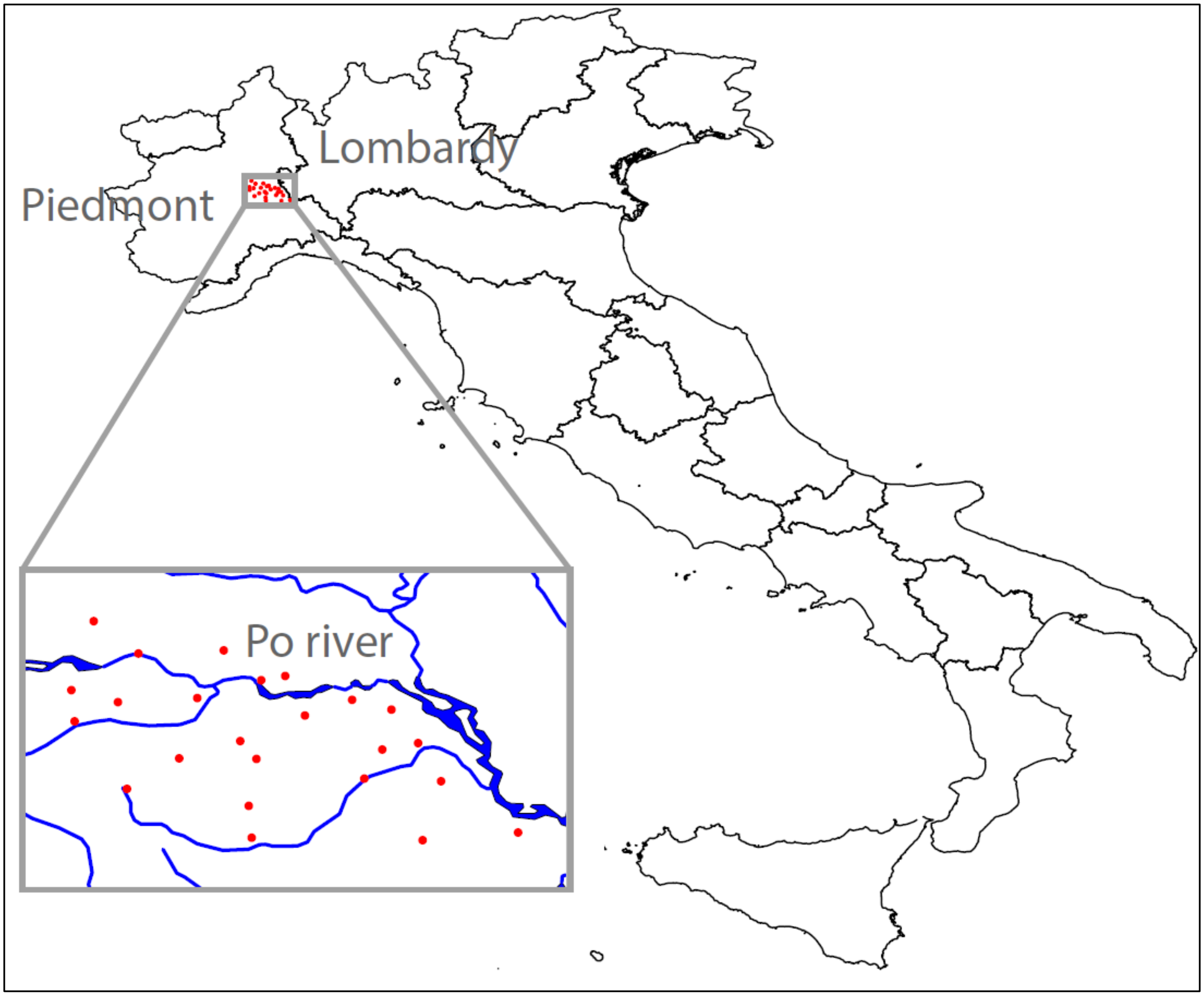

Figure G. Study area. The map shows the location of the 44 considered sites.

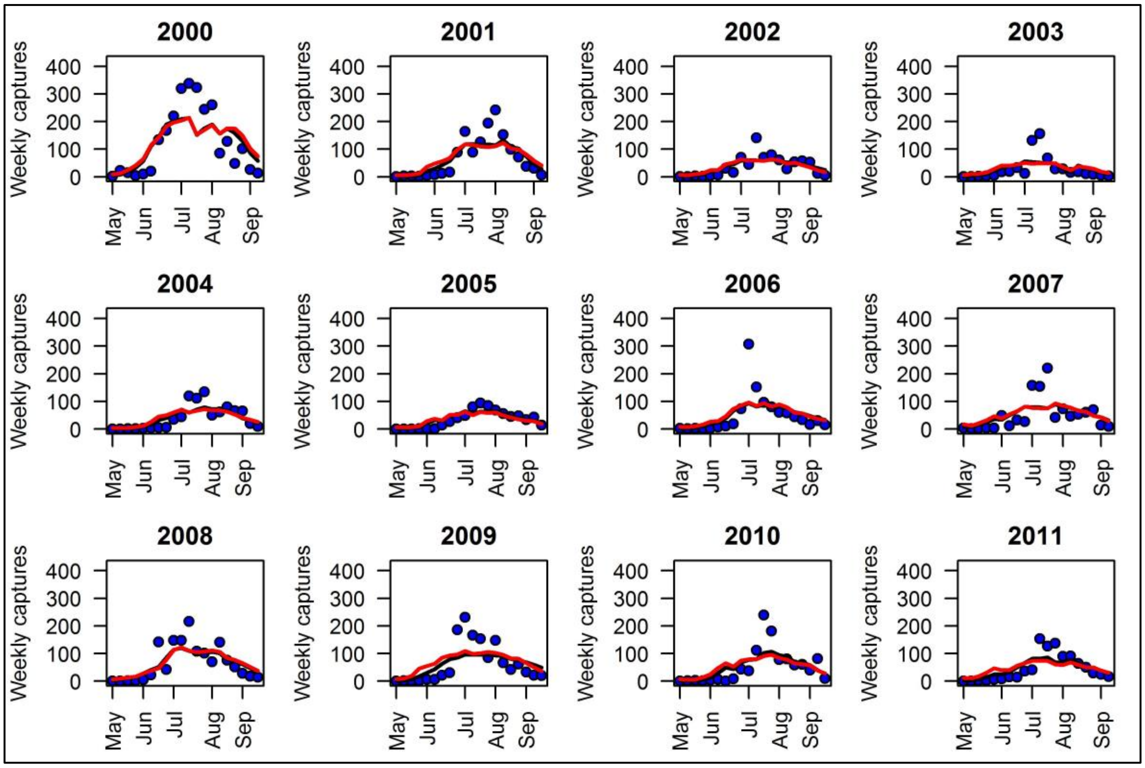

Figure H. Comparison of fit. Average number of weekly captured Cx. pipiens during the twenty-week survey period observed in Piedmont region from 2000 to 2011 (blue points) and median predictions by model simulation based on the estimated posterior distribution of free parameters by assuming α and β to be seasonal independent (red line) and by considering different α and β for each considered year (black line).

References

1. Loetti V, Schweigmann N, Burroni N. Development rates, larval survivorship and wing length of Culex pipiens (Diptera: Culicidae) at constant temperatures. J Nat Hist. 2011; 45(35-36): 2207–2217.
2. Chowell G, Diaz-Duenas P, Miller JC, Alcazar-Velazco A, Hyman JM, Fenimore PW, et al. Estimation of the reproduction number of dengue fever from spatial epidemic data. Math Biosci. 2007; 208(2): 571–589.
3. Poletti P, Messeri G, Ajelli M, Vallorani R, Rizzo C, Merler S. Transmission Potential of Chikungunya Virus and Control Measures: The Case of Italy. Roberts MG, editor. PLOSONE. 2011; 6(5): e18860.
4. Spielman A, Wong J. Environmental Control of Ovarian Diapause in Culex pipiens. Ann Entomol Soc Am. 1973; 66(4): 905–907.
5. Eirayah E, Abugroun N. Effect of Temperature on Hatching Eggs and Embryonic Survival in the Mosquito Culex-Quinquefasciatus. Entomol Exp Appl. 1983; 33(3): 349–351.
6. Kemeny JG, Snell JL. Finite Markov chains. New York: Springer-Verlag; 1976.
7. Ciota AT, Matacchiero AC, Kilpatrick AM, Kramer LD. The Effect of Temperature on Life History Traits of Culex Mosquitoes. J Med Entomol. 2014; 51(1): 55–62.
8. United States Naval Meteorology and Oceanography Command (2015) Naval Oceanography Portal: Data Services. Available: http://www.usno.navy.mil/USNO/astronomical-applications/data-services/data-services
